# Attachment, Bushfire Preparedness, Planning, and Response among Animal Guardians: A South Australian Case Study

**DOI:** 10.1371/currents.dis.f659ce48594ea47f5a20de03e9dfa43a

**Published:** 2018-08-02

**Authors:** Lisel O'Dwyer, Kirrilly Thompson

**Affiliations:** Senior Research FellowCentral Queensland University; Associate Research Professor, University of South Australia, City West Campus, Business School

## Abstract

Abstract Background: Animal ownership has been identified as a risk factor for human survivability of natural disasters. Animal guardians have been reported to react or act in ways that may put their own safety and that of emergency services personnel at risk when faced with a natural disaster. Recent research has suggested that this risk factor could be reconfigured as a protective factor, whereby desires to save animals from natural disaster harm could motivate increased planning and preparedness behaviours amongst animal guardians. However, there has been no research to determine if bushfire planning and response behaviours differ between pet owners with low and high attachment; and how the relationship may differ in relation to small or large animals. Methods and procedure: We investigated the relationship between people’s emotional attachment to different types of pets and their preparation and actions during the Pinery bushfire in South Australia in November 2015. Thirty-four people who were impacted by the fire participated in an online survey. Data were collected about their preparedness, planning and response behaviours as well as their animal attachment (high or low). Results: We identified 10 characteristics (behaviours, attributes, skills and beliefs) associated with high animal attachment scores, and eight associated with low animal attachment scores. Discussion: Our discussion of the differences in demographics, preparedness, planning and response characteristics of participants with high and low animal attachment confirms research suggesting that animal guardians take risks to save their animals during disasters. Our findings also support recent propositions that animal attachment and ownership could be used to increase the natural disaster preparedness and survivability of animal guardians. However, making sure that animal attachment functions as a protective factor requires active and effective intervention through education, behaviour change and social marketing strategies. Whilst our study is high in ecological validity, future research with larger samples sizes is required to determine the generalisability of our findings to animal owners and guardians in other locations, facing fires with other characteristics, especially for owners and guardians with low levels of attachment.

## Introduction

Animal ownership has been identified as a risk factor for human survivability of natural disasters. Animal guardians have been reported to react or act in ways that may put their own safety and that of emergency services personnel at risk when faced with a natural disaster 1^,^^2^^,^^3^^,^^4^. Recent research has suggested that this risk factor could be reconfigured as a protective factor, whereby desires to save animals from natural disaster harm could motivate increased planning and preparedness behaviours amongst animal guardians. Whilst the primary beneficiaries are humans and animals, improving the natural disaster survivability of animal guardians can also reduce demands on responders, emergency services and evacuation centres 5 ^,^^4^ ).

Most of the research on the impact of animals on human responses to natural disasters has focussed on the small animals that are most readily identified as pets, such as dogs and cats^6^^,^^7^^,^^8^^,^9. However, there are other larger animals with whom humans develop high levels of attachment, but who rarely share the same domestic spaces as humans. Often, small and large companion animals are present in a single household. A survey of 606 participants living in regional South Australia, for example, found that 74 percent of households were responsible for pets, of which 12 percent were responsible for horses or ponies and 7 percent were responsible for alpacas^10^. Another survey of horse owners in Australia determined that ‘the average numbers of [other] animals owned were two dogs, two cats, eight birds, two reptiles, 188 sheep, 27 goats or 45 ‘other’ animals’11. Attempting to evacuate from a fire threat with a horse is vastly different compared to a dog or a cat, and some households may be trying to evacuate both small and large companion animals – and not necessarily from the same property. Large companion animals are usually more difficult or even dangerous to handle and transport without specialised skills and equipment^12^ and emergency responders in Australia have noted that horse owners can be particularly difficult as they will do anything for their horses.^3^

Some of the reasons for the evacuation failure of small animal guardians have been practical, such as a lack of sufficient animal carriers or leashes^7^. From an emotional perspective, ‘animal attachment’ is often used to explain why people refuse to leave without their pets, or enter hazardous areas to ‘rescue’ them 7. It seems plausible that the degree of human risk taking to save animals would increase with the strength of the attachment. However, the implications of animal attachment for human responses to bushfires are not straightforward 13 and high animal attachment has not been found to be a predictor of evacuation^14^.

Still, there has been no research to determine if bushfire planning and response behaviours differ between pet owners with low and high attachment; and how the relationship may differ in relation to small or large animals. In relation to the preparedness stage of a natural disaster, there is a need to understand how attachment levels might be associated with animal identification, having evacuation kits, confidence administering first aid and perceptions of animals’ behaviours in response to fires. In relation to planning, there is a need to better understand how animal attachment characterises written plans and attention to fire warnings. In relation to response to actual fire threats, there is a need for ecologically valid data on how animal attachment impacts ultimate actions taken on catastrophic days, evacuating other people’s animals, evacuation without one’s own animals, perceptions of unnecessary evacuations and the driving rationale for research into the impact of animal ownership on human natural disaster survivability – risks people take to save animal lives.

We investigated the relationship between people’s emotional attachment to different types of pets and their preparation and actions during the Pinery bushfire in South Australia in November 2015.

## The Pinery fire and affected areas

As illustrated in Figure 1, the Pinery fire burnt 85,000 hectares in cereal cropping/grassland country known as the northern Adelaide Plains, located 80 km north of the Adelaide metropolitan area. The fire caused two human fatalities with 31 injured, destroyed 97 houses and destroyed or severely damaged 546 non-residential structures and 412 vehicles and pieces of machinery were. More than 50,000 poultry and 18,000 livestock (mostly sheep) also perished along with 120,000 tonnes of cereal crops worth up to A$30 million^15^ .

Townships and populated localities within or largely within the firescar include Alma (population 75), Barabba (117), Fischer (62), Grace Plains (54), Hamley Bridge (717), Linwood (48), Magdala (53), Morn Hill (17), Pinkerton Plains (76), Stockport (263), Templers (125), Wasleys (722) and Woolsheds (29), with a total population of about 2360^16^.

Nearby townships and localities and those partly within the firescar include Daveyston (75), Freeling (2214), Greenock (1087), Mallala (894), Nain (29), Owen (511), Pinery (102), Redbanks (182), Roseworthy (994), Stockyard Creek (20) and Tarlee (302), total population 6410^16^ . Local Governments for the affected and adjacent areas include Mallalla District Council, Clare and Gilbert Valleys Council, Light Regional Council and Wakefield Regional Council.

The socioeconomic profile of the affected and surrounding townships and localities is somewhat lower than for South Australia as a whole, with 17.1 percent of the population holding a bachelor’s degree or higher and 10 percent employed in professional occupations, compared to 32.9 and 20.2 percent for South Australia respectively^16^. Agriculture and manufacturing are the main industrial base (together employing 26 percent of workers, compared with 12.5 per cent for South Australia)^16^ although as a peri-urban area, a considerable percentage of the working population is likely to commute to Adelaide or other small regional centres.

## Method

****Survey design****

An online survey was targeted at pet owners affected by the Pinery fire, ie persons located within or near the fire. The survey was adapted from an extensive survey tool developed for use in post-fire event community taskforce research overseen by the Bushfire and Natural Hazards CRC^17^. Questions asking about specific preparedness actions relating to animals were informed by the literature on the topic^18^, as well as the experience and advice of industry partners from Horse SA, the SA CFS and RSCPA Qld. As a widely used and well-validated measure^19^^,^^20^^,^^21^ , the 23 item Lexington Attachment to Pets Scale (LAPS)^22^ was included to measure respondents’ attachment to their pets.

Most of the 130 questions were “tick box” format, including (up to) six questions with five-point rating scales and excepting seven questions requiring single word responses for each dog or horse owned or agisted. Twelve of the tick box options were multiple response, indicated by the instruction “tick all that apply” while the remainder required a single mutually exclusive response, eg “yes”, “no”, “don’t know” or “other”. If the “other” option was selected, respondents were able to provide optional explanatory detail. “Other” responses were coded to existing codes where appropriate. There were no unanticipated responses requiring additional codes. Not all questions were applicable to all respondents and respondents were automatically skipped to the next applicable question based on previous responses. The average time taken to complete the survey was 35 minutes.

The survey was constructed using the Qualtrics online survey platform www.qualtrics.com. Survey data were downloaded from the Qualtrics website in SPSS format and analysed in SPSS Version 23.

**Recruitment**

Residents of the Pinery area were invited to participate via a mailout of flyers to approximately 400 addresses in the affected areas which were targeted by the local postmasters who had personal knowledge and experience of affected streets, roads and households. The flyer explained the purpose of the research and directed householders to the online survey. Posters explaining the research with tear-off tags bearing the survey URL were displayed in local shops, post offices, community noticeboards and the Bushfire Recovery Centre located in the nearby town of Gawler. Short articles inviting affected residents to participate in the survey and including the survey URL were included in the local newsletter distributed by the Light Electoral Office (and endorsed by the Member for the electorate of Light), the Pinery Fire Recovery newsletter distributed by the SA and press releases sent to local newspapers and the four local governments with areas affected by the fire. The nearby Gawler Country Fire Service (CFS) also distributed flyers and promoted the survey through its various public activities, while one of the researchers gave a talk about the aims of the survey at a Fire Season Ready Family Expo held in Hamley Bridge, a township affected by the fire (see Figure 1). The project (and directions to the survey website) was discussed on ABC North and West and the survey information and link placed on the Central Queensland University media page. HorseSA promoted the survey to horse owners in the area through its mailing list and local social or interest clubs were tagged on Facebook with information about the survey and directions to the link. Participants were invited to provide their contact details if they wanted to be in a draw to win an Aud$50 voucher from Bonnetts Saddlery or PETStock.

**Response rate**

Despite the extensive recruitment efforts, responses to the survey were limited, with an initial response rate of ten percent, based on 58 responses from a population of approximately 600 pet-owning households. The estimated number of households with pets is based on the 920 households in the directly affected areas^16^ and an estimated pet ownership rate of 70% of households for a rural area in South Australia; the AMA^23^ cites 68% for total South Australia in 2016. After removing blank responses and responses indicating no consent, the number of cases was 34, for a response rate of six percent.

**Analysis**

Descriptive statistics (means, sums and percentages) were used to summarise the data. Differences between groups were compared using t tests for continuous data and Chi-Square (χ2) tests for categorical data. Differences in proportions for two independent groups were analysed using z tests. Correlations were measured using Spearman’s Rho and Kendall’s Tau where appropriate.

The data have been weighted to reflect the demographic composition of the population usually resident within and adjacent to the firescar using 2016 Census age and sex data^16^ .

The LAPS was scored by summing the scores for the 23 items on a 5 point Likert scale, after reverse scoring the two negatively worded items (allowing a possible range of 23- 115) and then converted to a percentage. Where there were missing values for some items, the percentage was based on the total number of completed items, with a minimum of three completed items.

The distribution of attachment to all pets in general was skewed toward the high end of the range with a mean score of 73 on the percentage scale (n=27). Due to the small number of cases and the location of a natural break between 60 and 69, 60 was selected as the cut off point demarcating high and low or lesser levels of attachment.

Figure 3 presents the mean number of animals owned by survey respondents using the total survey population as the denominator as well as the total number of owners of each species as the denominator. This means for example that on average, any Pinery resident is likely to own 2.6 horses, but the average number of horses owned by Pinery horse owners only, is 6.4. Horses dominate the large animal pet group on both measures, while the most commonly owned small animals are dogs, cats and chickens. The marked discrepancies between the means for rodents and ferrets, and birds, goats and sheep, shows that although these are not as frequently owned as dogs, cats and horses, those few respondents who do own them tend to have a large number of them in comparison with dogs, cats and horses.

**Inclusion of animals in survival plans**

Most participants (70% of respondents, n=24) had a bushfire survival plan of some kind (written or mental). All of these bushfire plans included animals. Fifty-one percent (n=12) included all animals in the household whilst 46% (n=11) included small animals only (excluding poultry). Those who included all animals had both large and small animals; the single exception had small animals only.

Respondents who had a written plan (n=10) had the largest number of animals with a mean of 19, compared to a mean of nine for those who had a plan in mind (n=14). Respondents who did not have a plan (n=5) also had a mean of nine animals.

A comparison of those respondents who did not include their pets in the plan (n=10) with those who did (n=24), showed that they tended to be much younger (85% were aged under 44, compared with 31% of those who did include their pets). They were more likely to be in casual or part-time work (86% vs 15%) and less likely to have vulnerable family members (children aged under 5, a person with a disability or a person over 60 requiring a carer) (7% vs 29%). There was no difference between these two groups in their ownership of large vs small animals.

**Animal attachment scores**

Almost one third (29%, n=8) of respondents who answered the attachment questions had low attachment levels ranging between 25 and 60 (X̅=45), and 69% (n=18) were classed as having high attachment (61 and above, X̅=92). An independent groups t test confirmed a statistically significant difference in attachment scores between these two groups; t(10.1-) = 7.4, p<.001, equal variances not assumed, (CI 32.5, 60.5).

The numbers are small with some missing data and so the results should be interpreted with caution, but with these caveats in mind, some indicative patterns emerge from Table 2. The species are listed in rank order of attachment with horses at the top and chickens at the bottom. The attachment levels for dogs, horses and cats are similar and it is possible that the true mean scores for cats and horses may differ from the numbers presented here due to the small numbers of completed responses.

The gender balance was heavily tipped towards males in the low attachment group (89% were male, n=8), biased toward females in the high attachment group (31% male, n=18) and also biased toward males amongst participants who did not respond to the attachment measures (75% male, n=8). Respondents with low attachment were much younger than the other two groups, with 89% (7 of 8) aged between 35-44, compared with 29% for the high attachment group (n=18) and 2% for the no attachment responses group (n=8). Seventeen percent (3 of 18) of highly attached respondents were aged 75 or older and there were no respondents in this age range in the other two groups.

Highly attached animal owners were more likely than low attachment animal owners to have a large number of animals in their care with a mean of nine animals in their care (ranging from two to 41) and a variety of small and large animals (48% had both large and small animals). Low attachment animal owners had a mean of three animals (ranging from two to nine) and only one of the eight had large animals. Respondents who did not complete any of the attachment questions had the most animals [mean of 23, (excluding one outlier with more than 30 rodents or ferrets)] and none had small animals only – all had both large and small animals.

An important aim of this study was to determine if and how preparations and responses to bushfire differ between pet owners with low and high attachment. Behaviours, attitudes, beliefs and skills are discussed below arranged around the stages of preparedness, planning and response. Most of the analyses address horses and other pets (mostly small animals) separately, given the different management needs of each group. Horses’ size, handling, behaviour and transport requirements and their management and evacuation require different responses from those of small animals (Thompson 2013). Other large animals (“lovestock”, i.e. pet pigs, sheep, goats, cattle etc) were not well represented in the sample so no clear patterns for this group could be established from the limited available data.

**PREPAREDNESS**

**Animal identification**

With the exception of cats, the attachment score for each species (i.e. dogs, horses and other pets as a group) is used as the attachment measure in the following analyses of animal identification, rather than the summary maximum attachment for all species. Maximum attachment for all species owned including cats is used as a proxy for cat attachment due to the low response rate for the cat attachment questions.

All respondents reported some form of ID, regardless of attachment level. Dogs owned by highly attached respondents more than twice as likely to have their dogs microchipped and have other forms of identification than low attachment respondents but there was no real difference between the two groups in the rate of council dog registration (z=0.77, p=0.44). Council registration was the most common form of identification, followed by microchipping, but rates of microchipping were low for both attachment groups. The use of other types of collar ID was very infrequent and only used by highly attached respondents (see Figure 4). Two respondents commented that their dogs were registered but that the dogs chew each other’s tags off. One of these respondents used microchipping and the other reported that one of her two dogs had a council tag but the other did not. Respondents who did not complete the attachment measures were most likely to have their dogs microchipped but least likely to have council registration tags for them.

Only one cat owner had a low attachment score and this respondent did not complete the identification questions (note that of the 22 cat owners, 3 completed the attachment questions and 21 completed the identification questions). The data presented therefore reflect the cat owners who completed the identification questions, regardless of attachment level.

Although 44% cat owners (n=9) had their cats microchipped, half (51%, n=11) had no form of identification for their cats at all. Almost none (one percent) had a collar ID and 13% (n=3) had other forms of identification such as photos or papers (Figure 5 ).

None of the respondents with horses had low attachment and so Figure 6 shows the ID choices for horse owners who are highly attached and those with no attachment score. Six of the nine (66%) highly attached horse owners’ horses were clearly branded. Five percent their horse(s) microchipped and two (18%) had other forms of identification such as photographs or papers. All respondents with horses described their horses as pets or retired, or for pleasure and/or recreation, rather than for breeding and/or racing. Respondents with no attachment score for their horses were much more likely to have horses with unclear brands and much less likely to have other forms of ID such as photos or registration papers. In total, almost all (99%) of horse owners had some form of identification for their horses.

Of the twenty-three respondents with animals other than dogs, cats or horses, three had low attachment scores. Twelve respondents without attachment scores completed the identification questions and their responses are included in this analysis.

Figure 7 shows that pets other than dogs, cat and horses were much more likely to have no ID and to be species that are difficult to individually identify, such as fish, rodents, frogs and reptiles and chickens. The species owned by the three respondents with low attachment were goats, sheep, birds, chickens and ducks, although the sheep had ear tags. The branded animals were a cow and a goat. In sum, species other than dogs, cats or horses are unlikely to have any form of ID, including some pet sheep, pigs, goats and cattle.

**Evacuation kits**

Counterintuitively, highly attached respondents were much less likely to have their small animal evacuation kits packed and ready to go (13%, n=18 vs 42%, n=5 for the low attachment group) but this rate should be viewed in the context of the small number of cases in the low attachment group and the type of pet. The low attachment group was more likely to have pet species for which evacuation kits (apart from pet food) were not relevant. Species such as fish, reptiles, rodents or budgies are generally already confined to portable tanks or cages and do not require leads, collars, ropes or items not already in their cages. Yet while 64% of the highly-attached respondents (12 of 18 respondents) had everything they would need, it was not actually packed and ready to go. Conversely, all respondents without attachment scores had small animal kits packed and ready to go (n=6).

The sole respondent with low attachment and a horse reported that they did not have a kit packed but had everything they would need. More than a third (41%, n=4) of highly attached respondents (n=9) did not have any items needed for an equine evacuation kit at all, while another 43% (n=4) said they had everything they would need but not packed and ready to go. All of the respondents with no attachment scores (n=5) had kits packed and ready to go.

As evacuation kits were generally reported as not being relevant to their types of pets, only three respondents from the low attachment group completed the question about items included in their small animal evacuation kit, precluding a statistically valid comparison with the other groups. However, the patterns within the high attachment group reveal two distinct clusters of items more and less likely to be included in the kit. The first cluster appears to be the basic essentials – all (n=8) packed enough cages or containers for all pets, 89% (7 of 8) of the high attachment group packed food for at least three days, 89% packed food bowls, feeders or dispensers, 88% packed leads or harnesses with identification tags and 75% packed water. The second cluster of less frequently packed items were items possibly perceived as non-essential, or impractical to include in a kit. These were vaccination certificates (27%) and dietary, medical or behavioural information (20%). A similar pattern was reported by respondents without attachment scores (n=6) with the exception that none packed food to last for three days.

There were insufficient responses to the question on items in horse evacuation kits to allow comparison (no respondents from the low attachment group and three from the high attachment group). All three high attachment respondents reported the inclusion of most items listed in the survey question in their horse evacuation kit (enough halters and leadropes, means for tying horses and water and feed buckets, woollen blankets and towels and wire cutters or a sharp knife, first aid items, and tools for identifying horses such as paints or tags). Only one of the three included a hose and identification in the form of ownership papers or other documents. All respondents in the no attachment score group (n=6) included all listed items except for identification in the form of ownership documents (this item was not selected by any of these respondents).

**Confidence administering first aid**

Half (50%, n=8) respondents with higher attachment (n=17) were moderately, very or extremely confident in small animal first aid and the rest were somewhat confident, but all of the six respondents in the low attachment group who answered this question were at least moderately confident. All of the five respondents in the no attachment score group were very confident. That is, level of confidence in giving first aid to small animals was inversely associated with level of attachment (τc = -.4, p =0 .02). Further, the no attachment score group (n=6)was significantly more confident in giving small animals first aid than either of the attachment groups, based on the residual values with Bonferoni adjustments (χ2 =20.9(6), p=0.002).

None of the low attachment respondents completed the question on confidence with basic first aid for horses, but three of the eight highly attached respondents who completed the question reported that they were not at all confident (43%). Thirteen per cent reported that they felt very or extremely confident. All of the respondents with no attachment score were very confident (n=6). A Chi Square test showed a significant difference in confidence levels between the high attachment group and the no attachment score group (χ2 = 7.82(3), p=.03).

**Perceptions of animals’ instincts in fires**

The small number of cases (n=6) in the low attachment group means that caution is advised in interpreting the comparison with the high attachment group (n=17), but it is worth noting that 88% of the low attachment group thought that animals have intuition about fire, while only 16% (3 of 17) of the high attachment group held this view. Two thirds (66%) of the high attachment group though that animals do not have any fire intuition and 19% did not know. Respondents with no attachment score (n=6) were also unanimous in their agreement that animals do have intuition about fire. The comments indicated that respondents from all groups felt that even if animals generally have intuition about fire, they recognised that there may be individual variation – i.e. some animals do have instinct or intuition and others do not, and that even where animal do have that instinct, they may be prevented from acting on it:

*“My dogs were all locked in runs so unable to go anywhere”;*

*“My horse just stood still and watched the fire spread across the paddock”;*

*“I think most would be just as lost as people”;*

*“Generally yes if they can escape - however individual personalities may impact e.g. my most nervous dog would have to depend on the other dogs to lead her to safety - she would just cringe somewhere or run in any direction.”;*

*“They will find shelter if they can escape”;*

*“Some do but fences etc limit it”;*

*“Yes, but may not be able to escape due to fencing”.*

## Planning

**Written plans**

Highly attached respondents (n=18) were actually less likely than low attachment respondents to have a written plan (5%, vs 30% of the low attachment group and 77% of the group with no attachment score [n=8]). Just over half (56%) of the high attachment group had no plan at all while the other two groups had either written plans or plans in mind. Forty four percent of the highly attached group had either a written plan or a plan in mind, compared with all of the low attachment group and 97% of the no attachment score group.

Familiarity with the area is known to influence propensity to plan (Smith, Taylor and Thompson (2015). Most respondents were long term residents; 70% of both attachment level groups had lived in the area for more than five years. Highly attached respondents were much more likely than lesser attached respondents to have lived in the area between 11 and 20 years (44% vs 2%) while respondents with no attachment score had lived in the area the longest, with 84% living there between 11 and 20 years.

**Attention to fire warnings**

All respondents (regardless of attachment level) indicated that they pay attention to fire warnings.

## RESPONSE

**Actions on catastrophic days**

The different attachment level groups acted differently on catastrophic days: most respondents listen for fire warnings although at 55% the low attachment group was least likely to do this (Table 3). Approximately half (51%) of the high attachment group also said they actually do nothing, even if they listen to the warnings. Highly attached respondents were much more likely to stay at home or close to home (37%) compared to low attachment respondents (0%) but virtually all respondents with no attachment reported stayed close to home. About one third (32%) of highly attached respondents reported getting their evacuation kit ready, but no low attachment respondents reported this action.

**Evacuating other people’s animals**

Four per cent (one case of 17) of the highly attached respondents evacuated pets that they did not have primary responsibility for, while 42% (n=2) of respondents in the low attachment group (n=6) did so. None of the no attachment score group evacuated others’ pets (n=5). Although approaching statistical significance, a Chi Square test showed no differences between the three groups (χ2=5.62(2), p=0.06).

Highly attached respondents were also less likely to evacuate other people’s horses (9%, n=1 of 17) compared with two of the five respondents in the low attachment group and none of the no attachment score group. A Chi Square test showed that the differences between the three groups are not statistically significant (χ2=5.36(2), p=0.07).

**Evacuation without animals**

One respondent in the low attachment group responding to this question (n=3) evacuated without their pets. None of the 17 high attachment respondents who answered this question evacuated without their pets, although three clarified their experience with the following comments:

*“I had to leave the chicken”;*

*“I wasn’t home”;*

*“My partner evacuated without our pets”.*

This last comment suggests that the respondent herself would not have evacuated without her pets if she had had the option and also that individual members within households may have different views on appropriate actions in bushfire situations.

None of the low attachment respondents answered the question about evacuating without their horses. Of the seven high attachment respondents who answered this question, four left their horses behind, two did evacuate with their horse/s, and one evacuated with some horses but had to leave two behind.

**Unnecessary evacuation**

No respondents in the sample felt they had wasted their time or money evacuating before finding out that their property was not under threat, but only one of the three low attachment respondents and two of the seven high attachment respondents who answered the question felt they had still done the right thing. None of the low attachment respondents felt that evacuating had been a good practice run for the future or felt relieved that they had evacuated, but two of the seven highly attached respondents thought it had been a good practice run and five of the seven (74%) felt relieved.

**Risk to personal safety**

No respondents in the low attachment group answered the question “did your actions to protect your small animals involve any personal risk to your own safety?” Only eight respondents in the high attachment group answered the question, of whom two reported that they risked their own life while acting to protect their small animals. By contrast, all of the six no attachment score respondents reported risking their lives to save their small animals. These different response patterns were significantly different (χ2 (2)=7.88, Fisher’s exact p=0.01. Only three of the high attachment respondents who risked their lives rated the level of risk; one judged it as high, one as medium and the last as low.

A somewhat different pattern emerges for respondents acting to protect their horses – six of eight highly attached respondents took risks to protect their horses, and four of six rated the risk as high. None of the low attachment respondents took personal risks to their own safety to protect their horses. All of the no attachment score respondents took high risks to protect their horses. There was no statistically significant difference between these two groups in their actions to protect horses (χ2 (2)=2.86, p=0.24).

## Discussion

**Participants**

The age and gender distribution of our sample is typical for both postal and online surveys 41^,^^42^ . The pet ownership rate for dogs and cats follows the usual pattern of being the most commonly owned species 23^,^^24^, although given the rural location, the rate for horses and chickens was comparatively higher than reported elsewhere. Excepting the greater representation of horses and the inclusion of other large animals, the distribution of pets by species is similar to the distribution reported by Taylor et al (2015:19)^4^ for respondents in both urban and rural areas.

All pet owners with plans included their pets in their plans - but not all pet owners had plans. At 30 percent, the percentage of respondents with no plan is much higher than the eight percent reported by Trigg, Smith and Thompson 13 for South Australian communities directly and indirectly affected by three large bushfires in January 2014 and the 19% reported by McLennan, Patton and Wright^43^ . The pattern of most respondents having only a mental plan (42%) rather than written (29%) is similar to Trigg, Smith and Thompson's findings^13^ (65% and 19% respectively), but six times higher than a national average of 5% found across several post-bushfire community surveys. The propensity for a mental plan rather than written was also held by the South Australian livestock producers surveyed by Smith, Thompson and Taylor^3^.

Whilst modest in size, this study of 34 pet owners affected by the Pinery fires identified 10 characteristics (behaviours, attributes, skills and beliefs) associated with high animal attachment scores, and eight which were associated with low animal attachment scores. As shown in Table 4, participants with a high animal attachment score were more likely than low attachment respondents to: be female, be older, own a horse, dog or cat; have more than the average number of animals, have dogs who are microchipped, stay home or close to home on potentially catastrophic fire risk days, evacuate with their horses, and take personal risks to their own safety to save horses but not small animals. Participants with low animal attachment scores were more likely to be male, younger, own animals other than dogs, cats or horses, not have an evacuation kit, be confident giving first aid to small animals and horses, perceive that animals have intuition or instincts about responding to fire, evacuate small animals and horses for which they did not have primary responsibility and not see their evacuation as good practice or feel relieved that they had evacuated unnecessarily.

The finding that highly attached respondents (who were also most likely to have horses) were less likely to have plans reflects the findings of Smith, Thompson and Taylor’s^3 ^study of emergency responders’ experiences of large animal rescue. ERs reported that most large animal owners had no or insufficient plans and that horse owners, in particular, are highly attached, which can equate to extreme emotions interfering with decision making and action during an emergency.

Gender may be a moderating factor in the relationship between attachment and level of confidence in giving first aid to both large and small animals, based on the well known relationship between gender and confidence^25^^,^^26^,^27^^,^^28^^,^^29^ However, an ordinal logistic regression analysis showed that although attachment level was a significant predictor of confidence in giving first aid (proportional odds ratio of 27.5, 95% CI,0.554-6.08,Wald (X2(1)=5.54, p=0.019), gender was not (odds ratio = 1:0.85, 95% CI,4.28 – 0.59, Wald X2(1)=2.2,p=0.137).

**Characteristics of participants with high animal attachment scores**

This study found that high animal attachment scores were associated with ownership of dogs, cats and horses. Most attachment studies focus on dogs and to a lesser extent cats. Comparative quantification of human attachment to horses and other small animals have been neglected in the literature, with most existing research addressing the attachment of horses to humans in the human-horse dyad^30^ or how humans interact with horses^44^. This finding confirms previous arguments that human-horse relationships share similar emotional attachment to companion animal relationships, despite horses not sharing domestic spaces of humans^31^.

Attachment levels for dogs, cats and horses were on par with other recent studies of pet owners experiencing natural disasters. Brackenridge et al^14^ reported an equivalent mean LAPS attachment score of 76 which compares favourably with the scores ranging from 70 for cats, 77 for horses and 71 for dogs in the present study. Taylor et al^4^ reported an average attachment score of 9.76 on a ten point scale for all species, or 97.6 in percentage terms compared with 73 in the present study, but this difference may reflect different study populations or instruments used to measure attachment.

Our study also found higher animal attachment scores reported by those with more than the average number of animals.

We found that older age groups and females were more likely to have high animal attachment scores. This is consistent with a study by Brackenridge et al^14^ who also used the LAPS and found a gender difference, as well as Bagley and Gonsman^32^ who reported a positive relationship between attachment and age. However, this finding could be peculiar to our sample, as Herzog’s review^33^ of twelve studies addressing gender differences in pet attachment found a small gender difference overall (invariably females feeling stronger attachment than males) although many studies found no difference^32^^,^^34^^,^^35^.

Unexpectedly, we found that those with high animal attachment scores were less likely to have prepared written bushfire survival plans. While this finding appears counterintuitive, it may be that high levels of attachment are associated with other factors negatively affecting propensity to plan, such as cognitive dissonance between currently enjoying and maintaining pets’ wellbeing and thinking about uncontrollable risks to their wellbeing, or gendered power structures within households determining if and how plans are made. Smith, Thompson and Taylor^3^ reported the comment of an emergency responder who also agisted other people’s horses: “many [horse owners] are [not] able to deal with the emotions involved in planning for their best friend ….. Some refuse to think about it and others over react". More detailed research is needed to unpack the possible psychological aspects of relationships between planning and attachment, and between planning and gendered household decision making.

The association between high attachment and low rates of planning does not necessarily undermine the idea that animal attachment can be used from a social marketing perspective to motivate preparedness 12^,^^36^^,^^45^. The question becomes how to acknowledge or account for the factors in an indirect relationship. Another important question is how to motivate pet owners with low attachment to prepare and act to include their pets and how to target them, given they are less likely to have ID for their animals, especially dog registration. Lower levels of attachment may also be associated with low rates of using veterinary care for their animals between disasters, which would preclude veterinary surgeries as an avenue for contact. Similarly, they may also be less likely to frequent pet supply stores or be involved in organised animal related clubs such as breed societies or dog obedience. More research is necessary to compare other animal-related leisure, shopping, or social behaviours and attitudes of highly attached and low attachment pet owners. Beverland, Farrelly and Lim^37^ argue for example that there are two main types of motivation for pet ownership – pets as companions to love, versus pets as toys, status markers and brands. Further, the different motivations of these two groups, which may be analogous to our highly attached and low attachment groups, affect their appreciation of the pet and the purchase of pet related paraphernalia. A more directive or prescriptive approach rather than relying on a strategy of providing information and raising awareness may be necessary for the low attachment subgroup of pet owners – if they can be reached. The prospect of prosecution under the Animal Welfare Act 1985 (SA) for failure to take reasonable action to protect or evacuate pets (admittedly difficult to police and prove either before or after a natural hazard) may also motivate some pet owners to give greater consideration to their pets.

Those with high animal attachment were more likely to remain at home or stay close to home on potentially catastrophic fire risk days. In relation to horse owners, further research may determine if this was because they were more likely to attempt to evacuate with horses, which is less practical than evacuating with smaller animals (Smith et al., 2014). Furthermore, it was not clear if attempts to evacuate with horses were directly related to those with higher animal attachment taking personal risks to their own safety to protect horses.

Evacuation drills or simulations for horses in specific areas have been reported to be effective overseas in identifying potential difficulties able to be addressed before an emergency occurs. While it may be logistically difficult to arrange on a community wide scale, it is possible for organisations such as pony clubs and adult riding clubs to conduct simulated evacuations. HorseSA reports that the protocol for a similar drill exercise has been available on the HorseSA website for more than three years but has never been downloaded (Fiedler, Pers. Comm. December 6, 2016). Perhaps such drills should be mandatory for all animal based organisations. Pet or horse owners who are not members of any animal related organisations can still be invited to participate in drills or given information via social media or other local community advertising such as posters and flyers from councils sent to post boxes.

Those with high animal attachment scores seemed more likely to engage in behaviours associated with responsible animal guardianship, such as preparing evacuation kits for small animals and horses as well as attaching council registration discs attached to their dogs’ collars and having microchipped dogs.

The identification rate for cats at 65% was low, even for highly attached cat owners, compared to the rate for dogs. The only formal or centralised means of identification for cats is via microchipping, which applied to only 42% of cats in this study – and these were cats owned by highly attached owners. The microchipping rate of cats in the general population of South Australia is currently unknown (but note that microchipping of dogs and cats in South Australia will be compulsory as from July 1, 2018^38^. The Australian Veterinary Association^39^ estimates that the microchipping rate for cats is 72% nationally (including those states without compulsory microchipping). Further, dogs have much higher rates of other forms of identification, mainly due to council dog registration requirements. In addition to microchipping, council registration for cats could be supported on the grounds of identification in emergency situations (including although not necessarily natural disasters).

Horse racing and equestrian sports’ requirements for identification and council dog registration requirements have benefitted emergency response and reunion after natural disasters. Other species may also benefit from stringent, centralised means of identification – particularly cats, but other small animals including birds, rabbits, ferrets, reptiles, tortoises and even fish can also be microchipped. At present, public awareness that these species can be microchipped is low. An animal identification promotion campaign may be useful. Such a campaign could be targeted to children (as well as the general adult population) to leverage their pester power and emotional ties to pets, even where parents’ attachment is low. Pressure from children may be particularly useful for species such as guinea pigs and rabbits, which are typically children’s pets. The ability to identify lost or stolen animals is likely to be the main motivation for microchipping such species but as with dogs and horses, will also be of benefit in the event of natural disasters. Note that microchipping is already mandatory for cats in all Australian States except South Australia, the Northern Territory and Tasmania.

**Characteristics of participants with low animal attachment scores**

Those with low animal attachment scores were more likely to be young and male. They were also more likely to own animals other than dogs, cats or horses. This pattern is consistent with the literature discussed above.

People with low animal attachment scores were more likely to be confident administering first aid, although most pet and horse owners reported that they felt very confident in administering first aid regardless of their level of attachment. However, this self-report survey cannot reveal if this confidence represents objective abilities in administering first aid.

Almost half (42%) of highly attached respondents reported that they were not at all confident treating horses. To confirm first aid abilities for all horse and pet owners, online information and other resources on first aid for animals during or after typical bushfires and other natural hazards can be developed. Such resources can be packaged for dissemination via social clubs or community organisations as well as groups such as dog obedience clubs, cat fancy clubs, riding clubs or pony clubs.

Those with high animal attachment scores were more likely to believe that animals have instinctive behaviours towards fires. This would seem to explain why they are less likely to evacuate with horses than those with high animal attachment scores. However, there is a need to understand if and how animal attachment might be related to beliefs about animal’s own fire-related instincts of ‘sixth sense’ for natural disaster survival. In particular, it might be necessary to systematically compare guardians’ beliefs with ethological knowledge about animal behaviour when facing bushfire threats or responding to other environmental hazards. The findings could have a significant impact on the ways in which owners of horses and other animals plan and prepare for and respond to natural disaster threat. Indeed, research on another fire in South Australia cases of horse guardians taking their cues to respond to a fire threat from their horses’ reactions and behaviours^46^.

Those with low animal attachment scores were more likely to evacuate pets and horses for which they did not have primary responsibility. The context for these evacuations requires further investigation, especially as the same group of people (with low animal attachment) are more likely to not have an evacuation kit and may have little or no experience with species such as horses, given their propensity to have small animals only (only one of eight respondents with low attachment had horses for example). This uneasy combination of apparent willingness to save other people’s animals without preparations may explain why this group was more likely to neither see their evacuation as good practice, nor feel relieved when their evacuation had been unnecessary (ie when in retrospect the fire threat did not seem to warrant evacuation). As noted elsewhere in relation to three major South Australian fires in South Australia,^46^ precautions may be required to ensure that evacuations are praised even if they may be described as unwarranted, lest they discourage pre-emptive evacuation in response to future fire threats.

**Limitations and further research**

The main limitation in this study was the small sample size and low response rate, with a possible bias toward more highly attached pet owners, who are more likely than low attachment pet owners to respond to a survey about pets. Whilst the strength of this small survey lies in the ecological validity of the Sampson Flat fire event, greater statistical power in future studies with a larger sample size may confirm statistical significance of some of the apparent differences evident in the current study and indicate their generalizability to other animal owners in regional areas of Australia.

Another limitation is the use of the LAPS for respondents who have many animals of the same or different species. Some respondents failed to complete all or any of the 23 item LAPS in this study, particularly cat and horse owners. The response rates for cats and horses may be an artefact of the survey structure, as the LAPS for cats and horses followed the measure for dogs, which were filled out by most dog owners. The LAPS may be too onerous or tedious for respondents with more than one or two pets to complete and thus may not be the best scale to measure attachment in rural areas where respondents are more likely to have multiple animals. Further, some of the LAPS items, such as “ I believe that ____ should have the same rights and privileges as family members” may not readily apply to species such as chickens, reptiles or fish, for most people.

Another issue requiring further attention is whether respondents who do not fill out attachment questions have similar, more or less attachment than respondents who do answer these questions, given that they formed 22% (n=8) of the 32 cases and also have more animals than either the high or low attachment groups. It is possible of course that the more animals a respondent has, the less likely they are to complete the 23 item LAPS due to the considerable effort required. Although the high attachment group also had large numbers of animals, the patterns of responses for this group sometimes resembled those of the low attachment group, while at other times were similar to the high attachment group.

## Conclusion

Despite our modest sample size, we were able to distinguish participants with high and low animal scores according to a total of 18 characteristics. Participants with a high animal attachment score were more likely to be female, older, own a horse, dog or cat; have more than the average number of animals, prepare small animal and horse evacuation kits, have dogs who are microchipped and wear a council registration disc on their collar; prepare written plans, stay home or close to home on potentially catastrophic fire risk days, evacuate with their horses, and take personal risks to their own safety to save animals. Participants with low animal attachment scores were more likely to be male, younger, own animals other than dogs/cats/horses, not have an evacuation kit, be confident giving first aid to small animals and horses, perceive that animals have intuition or instincts about responding to fire, evacuate small animals and horses for which they did not have primary responsibility and not see their evacuation as good practice or feel relieved that they had evacuated unnecessarily. Whilst our study is high in ecological validity, future research with larger samples sizes is required to determine the generalizability of our findings to animal owners and guardians in other locations, facing fires with other characteristics, especially with regard to owners and guardians with low levels of attachment.

## Competing Interests Statement

The authors have declared that no competing interests exist.

## Data Availability Statement

The data underlying this study have been uploaded to figshare and are accessible using the following DOI: 10.6084/m9.figshare.6297629.
